# A Case of Chemotherapy-Refractory “THRLBCL like Transformation of NLPHL” Successfully Treated with Lenalidomide

**DOI:** 10.1155/2018/6137454

**Published:** 2018-02-06

**Authors:** Mamatha Siricilla, Lydia Irwin, Andres Ferber

**Affiliations:** MD Anderson Cooper Cancer Center, Cooper University Hospital, Camden, NJ, USA

## Abstract

Nodular lymphocyte predominant Hodgkin lymphoma (NLPHL) is a subtype of nonclassical Hodgkin lymphoma (HL). It resembles non-Hodgkin lymphoma (NHL), by expressing classic B cell markers such as CD20 and CD79a however lacks definitive HL markers (such as CD15 and CD30). T cell histiocyte-rich large B cell lymphoma (THRLBCL), on the other hand, is a distinct entity classified under NHL and considered a variant of diffuse large B cell lymphoma (DLBCL). NLPHL can look morphologically and immunologically similar to THRLBCL and often poses a diagnostic challenge. Neoplastic cells in both NLPHL and THRLBCL express B cell markers and are typically scattered in a background of reactive cells. The two major differences are the background cell type and the morphologic pattern. Despite having a phenotypic resemblance, they have distinct biologic behavior and clinical course. NLPHL typically has an indolent course, and THRLBCL has an aggressive course. Hence, differentiating these two entities is critical not only for prognosis but for treatment purposes. Of note, NLPHL has a small risk of transformation to an aggressive lymphoma such as THRLBCL.

## 1. Introduction

Here We present a case of NLPHL with THRLBCL like transformation. We used single agent lenalidomide, after failing more than four lines of conventional combination chemotherapy. Using this treatment, we were able to achieve a complete clinical and radiological response (CR).

Based on our experience with this rather rare case presentation and clinical course, we want to highlight the diagnostic challenge in these two entities at de novo presentation and with transformation. Treatment of NLPHL and THRLBCL as individual entities in the relapsed refractory setting is challenging. This case highlights an example of refractory NLPHL with THRLBCL-like transformation at relapse and presents lenalidomide as a promising treatment option, a drug not typically used in this setting.

## 2. Case Presentation

A 35-year-old gentleman first presented with left axillary lymphadenopathy in 2010. He was subsequently lost to follow-up for approximately two years until February 2012. At this point, he was noted to have waxing and waning lymphadenopathy in the left axilla and a 100 lbs unintentional weight loss. PET-CT scans showed diffuse lymphadenopathy involving the left upper chest wall, left axillary (SUV 15.1), left upper neck, right iliac (SUV 16.2), and inguinal regions. He underwent biopsy of the axillary lymph node (March 2012), and pathology showed an atypical lymphoid infiltrate of medium-sized cells. By immunohistochemistry (IHC), the cells were positive for CD20, BCL6, and CD45 and negative for CD15 and CD30. Large numbers of CD3-positive small lymphocytes were noted in the background along with CD57 cells surrounding the tumor cells, which was consistent with the diagnosis of NLPHL. A bone marrow biopsy was performed which was negative for involvement with lymphoma. He received chemotherapy with six cycles of rituximab (R), cyclophosphamide (C), Adriamycin (H), vincristine (O), and prednisone (P) and achieved partial response only. During that time, there were multiple delays in treatment due to complications including a pulmonary embolism (June 2012), necrotizing fasciitis, and septic arthritis with group A *Streptococcus*. Due to the partial response, he was subsequently treated with second-line therapy using ifosfamide (I), gemicitabine (GE), and vinorelbine (V) with rituximab (R) for four cycles. PET-CT scan performed at completion of this in February of 2013 showed near resolution of all the previous FDG avid lesions, except for a small amount of metabolic activity in the left axilla with an SUV of 2.5. At that time, it was recommended that the patient should undergo autologous stem cell transplant; however, he failed to follow-up with his appointments. Subsequently he received radiation for residual disease in the left axilla (August 2013).

Following radiation therapy, the patient felt clinically well until July 2014, which was nearly a year since his last treatments and four years from his initial presentation. Routine surveillance PET-CT in July 2014 revealed recurrent disease with diffuse lymphadenopathy including new hypermetabolic lesions in the retroperitoneum and cardiophrenic regions, the largest of which was in the peri-pancreatic area measuring 12.5 × 7.0 × 4.0 cm, with an SUV of 12.5. Fine-needle aspiration with endoscopic ultrasound of the peripancreatic lymph nodes was nondiagnostic. He was lost for follow-up and presented several months later with night sweats, fevers, weight loss, and dyspnea on exertion. PET-CT scan in December 2015 showed splenomegaly and progressive diffuse lymphadenopathy. Excisional biopsy of the left inguinal lymph node revealed a partially intact capsule; architecture was effaced by a nodular and diffuse lymphoid infiltrate of predominantly small mature lymphocytes with admixed large atypical lymphocytes. The large atypical lymphocytes were multilobulated with vesicular chromatin and nucleoli that ranged from multiple intermediate- to large-sized central nucleoli, consistent with lymphocyte predominant cells. The large atypical cells were noted in the diffuse regions of the lymph node but were more numerous in the nodules. Immunohistochemical stains of the large atypical cells were positive for CD45, CD79a, PAX5, BCL6, MUM1, OCT2, BOB1, and IgD and negative for CD30, CD15, CD10, and ALK. A subpopulation of B cells was positive for CD20. Scattered immunoblasts were positive for CD30. EBV (Epstein–Barr virus) in situ hybridization was negative. Of note, there was a marked T lymphocyte infiltrate in a background of predominantly CD4 + T lymphocytes. There were rosettes formed by CD3-, CD4-, and PD-1-positive cells around scattered large atypical cells. There were nodules with expanded follicular dendritic cells highlighted by CD21 and CD23. A subset of T lymphocytes was CD57 positive, and scattered CD163-positive histiocytes were also noted. The lesions had features of both nodular lymphocyte predominant Hodgkin lymphoma and T cell histiocyte-rich B cell lymphoma. Positivity of immunoglobulin D and the presence of follicular structures still favored a diagnosis of NLPHL.

**Figure 1:**
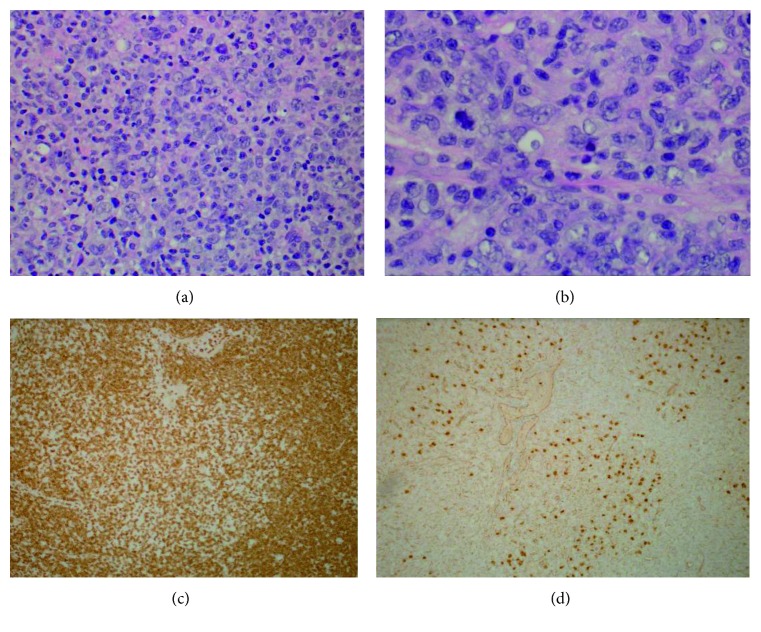
Haemotoxylin and eosin (H&E) stain (a) at 400x magnification showing a diffuse appearing infiltrate composed of scattered large atypical cells in a background of smaller lymphocytes and histiocytes and (b) at 600x magnification showing lymphocyte predominant cells. (c) CD3 stain highlights abundant background T cells and shows a hint of nodularity (center) shown at 100x magnification. (d) Large tumor cells are strongly positive for PAX5. Note the presence of the nodule of tumor cells in the center.

**Figure 2:**
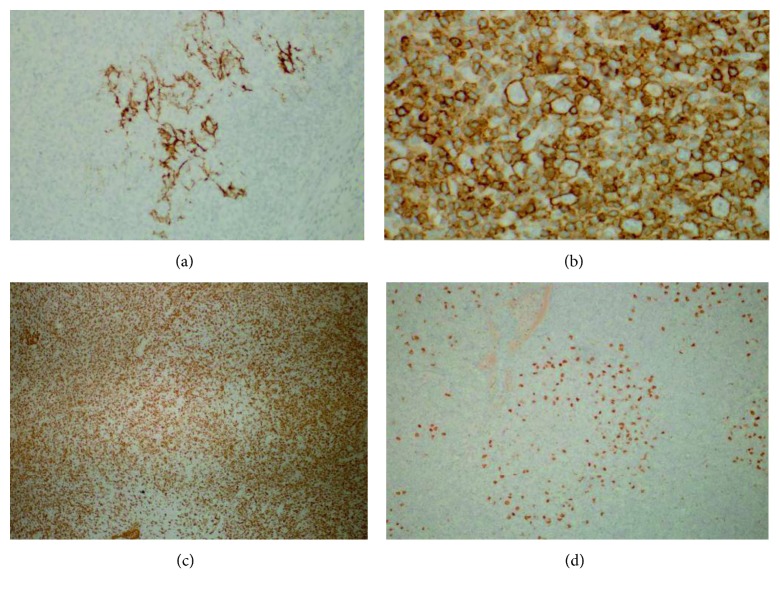
(a) CD21 stain highlights rare dendritic meshworks shown at 200x magnification. (b) The large tumor cells are strongly positive for CD45 (this is essential in excluding classical Hodgkin lymphoma from the differential diagnosis; both NLPHL and THRLBCL will be CD45+) shown at 400x magnification. (c) CD163 stain highlights abundant background histiocytes shown at 40x magnification. (d) Large tumor cell nuclei are strongly OCT2 positive. Note the presence of tumor cells in the center shown at 100x magnification.

He was started on third-line therapy with rituximab (R), ifosfamide (I), carboplatin (C), and etoposide (E) chemotherapy in April 2016, and after two cycles of therapy, he was found to have no response. His course was complicated by systemic cryptococcal infection, and chemotherapy was held. A repeat PET-CT scan in June 2016 revealed extensive disease involving the lymph nodes, bone marrow, and spleen. Hypermetabolic lymph nodes in the bilateral neck level II/V had an SUV of 13.0, the gastrohepatic ligament had an SUV of 14.4, the right external iliac had an SUV of 21.4, and the mesentery had an SUV of 19.3. Hypermetabolic lesions were also noted in the sternum, right proximal femur, and clivus with a maximum SUV of 14.0. The spleen was markedly enlarged and diffusely hypermetabolic with a focal hypermetabolic lesion in the posterior region with a maximum SUV of 13.0. The liver was also enlarged with multiple hypermetabolic lesions, the largest of which was in the posterior right hepatic lobe with a maximum SUV of 9.0, all consistent with lymphoma involvement. Overall Deauville score was reported to be four. A repeat biopsy (right cervical lymph node) was performed. Pathology from this specimen showed extensive effacement of the normal architecture by an atypical predominantly diffuse but focally vague nodular polymorphous lymphoid infiltrate composed of a mixture of small, medium, and large cells. Specifically, many scattered large atypical cells are distributed throughout the infiltrate within a background of abundant small- to medium-sized mature lymphocytes and histiocytes. The large atypical cells possessed moderately abundant pale cytoplasm and large markedly irregular/lobulated nuclei with coarse vesicular chromatin. Scattered mitotic figures and occasional multinucleated forms were present. No significant eosinophil or plasma cell infiltrate was appreciated. While the majority of the infiltrate exhibited a diffuse growth pattern, a focal area of nodular growth was also present. Immunohistochemical stains revealed that the large atypical tumor cells were B cells, positive for CD45, PAX5, CD79a, BCL6, OCT2, MUM1, CD30 (minor, weak subset), and EMA (very rare cells, weakly positive) and negative for CD20, CD10, CD15, and ALK. CD3- and CD163-positive stains highlighted an abundant background of T cells and histiocytes, respectively (see Figures 1 and 2 for pathology slides). A subset of lymphocytes was CD57 positive without obvious rosette formation. CD21 and CD23 stains were almost negative, highlighting only rare residual dendritic meshworks of follicular structures. An additional immunostain for Ki 67 highlighted a high proportion of tumor cells (greater than 75%). In situ hybridization for EBER (Epstein–Barr encoding region) was negative. Overall, the lesions had features of both NLPHL and THRLBCL. Given the focal nodularity and the patient's prior history of NLPHL, diagnosis was best classified as NLPHL-THRLBCL-like (old terminology) now updated in the new 2016 WHO classification as “THRLBCL-like transformation of NLPHL” (see Figures 1 and 2 for pathology description).

**Figure 3:**
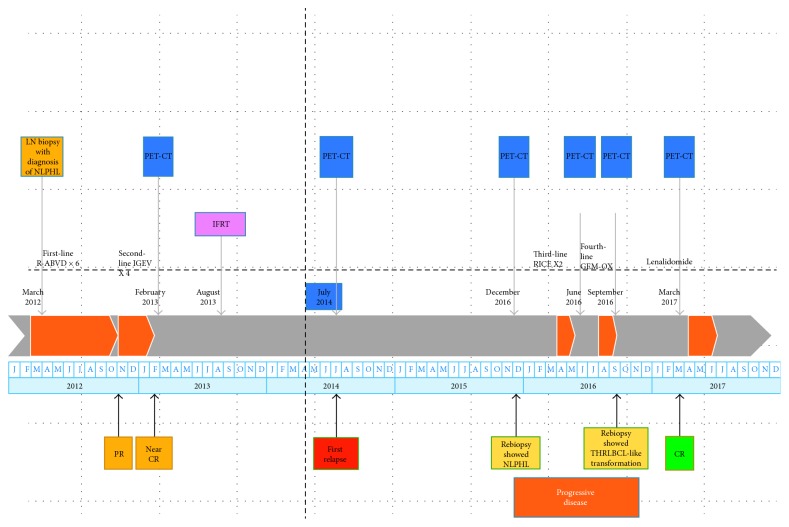
Treatment schema.

The patient subsequently received fourth-line treatment with two cycles of gemcitabine/oxaliplatin (August 2016) and demonstrated further progression of the disease. A PET-CT scan (September 2016) revealed new hypermetabolic adenopathy, with greater than thirty hypermetabolic lymph nodes throughout the body. There were also extensive hypermetabolic osseous metastases throughout the skull, ribs, spine, pelvis, and proximal femur. There were also new hypermetabolic lesions in the liver. The Deauville score was five (see Figure 3 outlining his treatment schema).

Further chemotherapy with regimens such as DHAP (dexamethasone, high-dose ara-C (cytarabine), and cisplatin) was planned. At this time, the patient felt poorly with prior chemotherapy regimens. His course was complicated by systemic cryptococcal infection as well as the cumulative toxicities of the prior therapies. Due to these reasons, additional chemotherapy was withheld. Tumor cells were tested for PD-L1 expression and it was negative.

**Figure 4:**
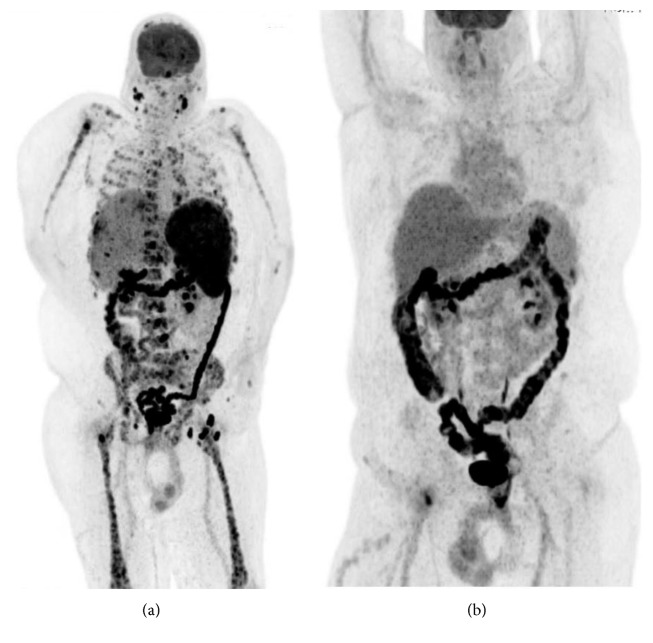
PET-CT images: 3-dimensional MIP images.

**Figure 5:**
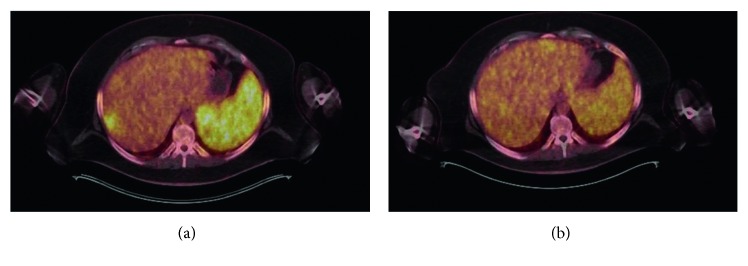
Fused PET-CT images: abdominal window showing resolution of liver and spleen lesions (a) prior to lenalidomide use and (b) after lenalidomide use.

**Figure 6:**
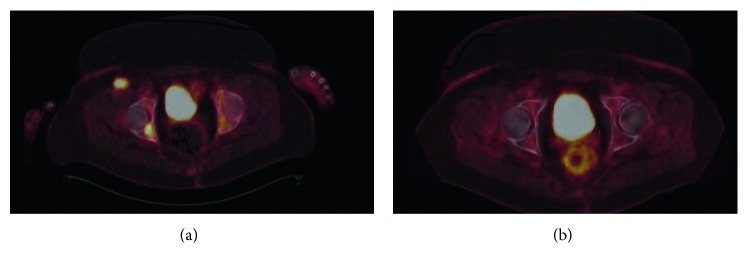
Fused PET-CT images: bone window showing resolution of bone lesions (a) prior to lenalidomide use and (b) after lenalidomide use.

Lenalidomide was initiated in November 2016 (25 mg daily for 3 weeks on and 1 week off schedule). The patient experienced rapid clinical response with resolution of fevers, night sweats, and subsequent weight gain. PET-CT scan performed five months after lenalidomide initiation showed resolution of the previously noted lesions in the bones, lymph nodes, and liver (see Figures 4–6 for PET-CT image comparison showing resolution of the disease).

## 3. Discussion

NLPHL is a rather uncommon subtype of nonclassical Hodgkin lymphoma (HL) constituting only 5% of all HL cases [1]. It shares morphologic resemblance to the lymphocyte predominant type of classical HL, hence classified under this category [2]. However, this disease entity is very distinct from classical HL. Specifically, the cells have a germinal center B cell phenotype characterized by positivity for CD20, CD79a, and BCL6 cells, immunoglobulin expression, J chain, and epithelial membrane antigen and negativity for classic markers for HL such as CD15 and CD30 [3–6].

Immunophenotypically, NLPHL is distinguished by the presence of large neoplastic cells (lymphocytic predominant cells) in a nodular distribution, with scattered nonneoplastic small B cells and T cells and the presence of a meshwork of dendritic follicular cells. CD3-, CD4-, and CD57-positive small T cells form rims around the large neoplastic B cells [2].

Immunophenotypically, THRLBCL is distinguished by the presence of large neoplastic cells in a diffuse pattern (as opposed to the nodular pattern seen in NLPHL). In THRLBCL, malignant B cells are scattered on a background of small nonneoplastic CD8-positive T cells and histiocytes, with rare small B reactive cells, which are different from the reactive small T cells, and abundant reactive B cells seen in NLPHL. Neoplastic B cells constitute only 10% of the infiltrate in THRLBCL [7]. T cell rosettes, small B cell lymphoid aggregates, and nodular infiltrate with a follicular dendritic cell meshwork typify NLPHL, which are absent in THRLBCL. PU.1, a transcription factor deemed necessary for B cell proliferation, is noted to be expressed in all cases of NLPHL but reduced or absent in THRLBCL [8]. Genomic imbalances were also noted to be more frequent in NLPHL than in THRLBCL [9]. The later described distinctions argue against direct evolution of one disease into another.

Due to similarities in the phenotype, both NLPHL and THRLBCL can look morphologically and immunologically alike [10], thereby making the diagnosis quite challenging [11–13]. Because these two entities have distinct biologic behavior, clinical course, and aggressiveness, it is critical to differentiate them prior to making therapeutic decisions. NLPHL typically presents with localized disease, involving the peripheral lymph nodes. Spleen and extranodal disease at presentation is rare [14], and the clinical course is indolent [15]. THRLBCL is aggressive and typically presents in advanced stages, with nearly 50% of cases having bone marrow, spleen, and liver involvement [16]. As such, THRLBCL is associated with poor outcomes [17–20]. Traditional therapy for stage I/II NLPHL is full nodal excision followed by either a “watch or wait strategy” or localized radiation [21–25]. The challenging part of NLPHL is the relapsing nature of the disease and the potential risk (3–7%) [14] of transformation into a large B cell lymphoma, sometimes even decades later [26]. THRLBCL is one of the most common types of large B cell lymphoma, seen as a result of transformation of NLPHL [27]. Advanced stages of NLPHL are typically treated with ABVD similar to HL, with the exception of the addition of rituximab due to CD20-positive cells. The treatment approach for THRLBCL is similar to DLBCL and typically involves regimens such as R-CHOP. In a subset of NLPHL cases that present with B symptoms and/or abdominal involvement, R-CHOP is favored over ABVD due to the risk of occult transformation to large cell lymphoma [1]. Stage-adjusted overall survival rates for NLPHL are over 90% and over 50% for THRLBCL [15]. In contrast, these survival rates reiterate the importance of differentiating these two diseases in order to make appropriate therapeutic decisions [28]. There have been interesting observations reported in the literature where these two entities were reported to be present concomitantly or seen on subsequent biopsies [18, 19, 26]. NLPHL is believed to be derived from a precursor lesion described as “progressive transformation of germinal center B cells” (PTGC). Thus, it may be possible that this represents a continuum of a single disease or a natural course of diseases from PTGC to NLPHL and eventually THRLBCL [26, 29].

In a large prospective study conducted by the British Columbia Cancer Agency, the risk of transformation of NLPHL into large cell lymphoma was reported to be 7% and 30% in 10 and 20 years, respectively. This risk is higher in patients with advanced-stage disease at presentation and if there is splenic involvement. Splenic involvement is an independent predictor for transformation. It is seen in almost all cases of NLPHL transformations (close to 80%), unlike de novo large cell lymphoma where the chances of spleen involvement were only 20%. It is also recommended that patients with findings suggestive of splenic involvement at the time of diagnosis of NLPHL should be strongly considered for splenectomy to rule out the concurrent presence of DLBCL and/or THRLBCL [9].

Findings of the pathology in our case at the time of progressive disease after four lines of therapy (see Figure 3 for treatment schema) had features of both NLPHL and THRLBCL, with diffuse infiltrates but retained focal nodularity, rare follicular dendritic cells, and absent T cell rosettes. The presence of even one nodular lesion in the background of TCHRLBC will exclude the diagnosis of primary/de novo THRLBCL as per the WHO criteria [30]. In this particular case, the patient's prior history of NLPHL and subsequent biopsy showing both NLPHL and THRLBCL features best fit the diagnosis of NLPHL-like THRLBCL or “THRLBCL-like transformation of NLPHL” as per the updated 2016 WHO recommendations [31]. Literature review revealed few cases similar to ours with NLPHL-like areas in THRLBCL [14, 32] and these cases were either treated with R-ABVD or R CHOP. There was one case with discordant presentation with NLPHL in a lymph node and THRLBCL in the bone marrow [29]. Treatment of relapsed cases with large cell transformation is similar to DLBCL, typically using standard salvage chemotherapy followed by high-dose chemotherapy and autologous stem cell transplant [1].

To the best of our knowledge, there is no published literature for treatments of relapsed-refractory THRLBCL-like transformation of NLPHL. There is also no case reported indicating the use of lenalidomide for this disease entity.

Lenalidomide is known to be an active agent in heavily pretreated NHL [33, 34]. It had been successfully studied in the relapsed-refractory setting in various types of NHL such as mantle cell lymphoma [33], follicular lymphoma [35], diffuse large B cell lymphoma [36], and T cell lymphoma [37]. The FDA approved lenalidomide for relapsed mantle cell lymphoma after 2 lines of therapy [38]. The use of lenalidomide in newly diagnosed aggressive B cell lymphoma, particularly in nongerminal center-derived cases, has been reported in combination with R-CHOP (R2-CHOP) and has been shown to achieve objective response rates (ORRs) of 90–100% and CR of 77%–86% [39–42]. In addition, lenalidomide as monotherapy in relapsed refractory cases of NHL was shown to have an ORR of 35–28% [33, 34]. The benefit was much higher in nongerminal center-derived subtypes (ORR 52.9%) than in germinal center-derived subtypes (ORR 8.7%) [43]. The response rate in our case was nearly 100%, which is particularly exciting, given that this patient had previously failed four lines of treatment.

Lenalidomide acts by blocking tumor growth and survival by direct tumoricidal and immunomodulatory actions. Lenalidomide also modulates tumor cell microenvironment and stimulates activity of cytotoxic T and natural killer cells [44]. It causes inhibition of nuclear factor kappa B, leading to cell cycle arrest and tumor cell death [45]. Interestingly, the nongerminal center subtype of DLBCL was noted to have a high expression of NF kappa which might explain higher response rates in these subtypes [38]. In the relapsed-refractory setting, patients are heavily pretreated, and cumulative toxicities from prior treatments often become a barrier for additional chemotherapy.

Lenalidomide, on the other hand, has a highly manageable toxicity profile, making it a viable treatment option in certain circumstances. The most frequent reported grade 3-4 adverse events include neutropenia (43%), febrile neutropenia (5%), thrombocytopenia (28%), fatigue (7%), skin rash (23%), diarrhea (6%), and risk of thromboembolism. One downside of lenalidomide is the potential risk for second primary malignancies (incidence rate of 3.98%) [46]. The risk and benefits of such a drug should be discussed, especially when using in younger patients.

Based on our clinical experience with this case and the established activity of lenalidomide in NHL, we hope that the use of lenalidomide will expand into a viable option for NLPHL and/or THRLBCL-like transformation of NLPHL.

It would be interesting to design clinical trials using lenalidomide in NLPHL and THRLBCL-like transformation cases, when refractory to traditional treatments. Another area of research interest would be using lenalidomide as an upfront therapy to avoid exposure to potentially toxic chemotherapy combinations.
